# Adnexal torsion in the third trimester of pregnancy: a case report and diagnostic value of MR imaging

**DOI:** 10.1186/s12880-020-00422-1

**Published:** 2020-02-17

**Authors:** Wei Bai, Xiao Xu, Haizhu Xie, Chunjuan Sun, Kaili Che, Meijie Liu, Jing Liu, Yinghong Shi, Heng Ma

**Affiliations:** 1 Yuhuangding Hospital, Qingdao University School of Medicine, No. 20, Yuhuangding East Road, Yantai, 264000 Shandong Province China; 2 Weifang Medical University, Weifang, Shandong Province China; 3 Binzhou Medical University, Yantai, Shandong Province China

**Keywords:** Adnexal torsion, Magnetic resonance imaging, Pregnancy, Third trimester

## Abstract

**Background:**

The torsion of normal adnexa is rare during pregnancy, especially in the third trimester. Nonspecific symptoms and signs as well as the limitations of ultrasound (US) make the diagnosis difficult, resulting in the loss of adnexa and fetal compromise. The magnetic resonance imaging (MRI) features of the torsion of normal adnexa are not classically described during pregnancy and only reported in a few cases. We find some different MRI features of the torsion of normal adnexa in late pregnancy and its diagnosis and treatment values are discussed in our report.

**Case presentation:**

A 27-year-old woman at 31 + 5 weeks’ gestation presented to the emergency department with a three-day history of the left lower abdominal pain. US discovered a mass of 87 × 61 mm in the left abdomen, but did not show whether the mass originated from the left ovary or the uterus. MRI showed the left ovary was increased in size to 82 × 42 × 85 mm with peripheral follicles. On fat-suppressed T2-weighted images, the signal intensity of the lesion was significantly decreased compared with the right ovary. The adjacent fallopian tube was found to be thickened. The radiologists diagnosed ovary infarction secondary to adnexal torsion. With the provisional diagnosis of adnexal torsion, the patient was taken to surgery. The left adnexal torsion was found during surgery. There was extensive hemorrhage and necrosis, so a left salpingo-oophorectomy was performed. The histopathology confirmed an extensively hemorrhagic fallopian tube and ovary with partial necrosis.

**Conclusion:**

We believe MRI is helpful where US is indeterminate in diagnosis of the torsion of normal adnexa in advanced pregnancy. We found that aside from hyperintensity on fat-saturated T1-weighted images, the low signal intensity on T2-weighted images can also reflect adnexal hemorrhage in conjunction with the torsion of normal adnexa.

## Background

Adnexal torsion refers to the total or partial twist of the ovary around its vascular axis and can result in vascular compression and necrosis. A total of 13.7% of all adnexal torsion episodes are found in pregnant women [1]. Adnexal torsion occurs more frequently in the first and early second trimesters than in the third trimester [2]. The nonspecific clinical symptoms of adnexal torsion and the anatomical and physiological alterations in advanced gestation complicate the diagnosis of adnexal torsion. Although ultrasound (US) is the accepted mainstay modality for adnexal torsion, it is extremely limited for visualization of the ovaries in women in their second and third trimesters of pregnancy [3]. These factors can delay the diagnosis of adnexal torsion and surgical management [4], resulting in the loss of adnexa and fetal compromise.

Magnetic resonance imaging (MRI) is a safe and effective method for making diagnosis in advanced gestation [3]. MRI can have a role where US is indeterminate. We present a case of the torsion of normal adnexa in advanced gestation. The MR imaging features are not classically described during pregnancy and only reported in a few cases. Compared with several previous case reports [5–7], some different MR imaging features of adnexal torsion in late pregnancy are discussed in our study as well as its diagnosis and treatment values.

## Case presentation

A 27-year-old woman at 31 + 5 weeks’ gestation (gravida 0 para1, G0P1) presented to the emergency department with a three-day history of the left lower abdominal pain. The pain was constant and getting progressively more severe. She did not complain of nausea or vomiting. There were no history of urinary symptoms, fevers, vaginal bleeding or uterine contraction. Ovarian hyperstimulation therapy was not performed. The patient’s ovaries were noted to be normal on ultrasound prior to the start of pregnancy.

**Fig. 1:**
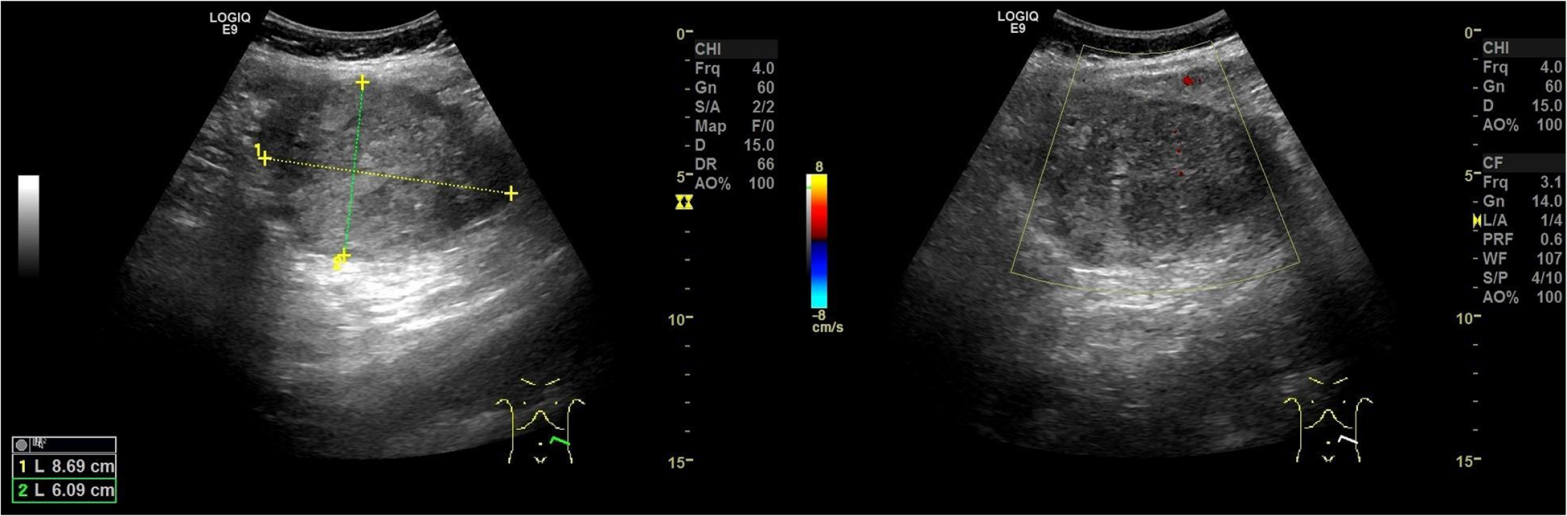
Ultrasound showed a heterogeneously hypoechoic mass in the left abdomen and Doppler revealed a lack of perfusion within the mass

On examination, the patient’s vital signs were stable. Abdominal palpation revealed a tender abdomen on the lower left side without signs of peritoneal irritation. The uterus was enlarged corresponding to 31 + 5 weeks, and the cervix was closed with a little discharge. An obstetric US demonstrated the fetal parameters that corresponded to gestation with normal amniotic fluid and fetal activity. A heterogeneously hypoechoic mass of 87 × 61 mm was discovered in the left abdomen adjacent to the left uterine border with unclear boundaries, which contains several non-echoic millimetric cysts. Doppler revealed a lack of perfusion within the mass (Fig. 1). US did not show whether the mass originated from the left ovary or the uterus.

**Fig. 2:**
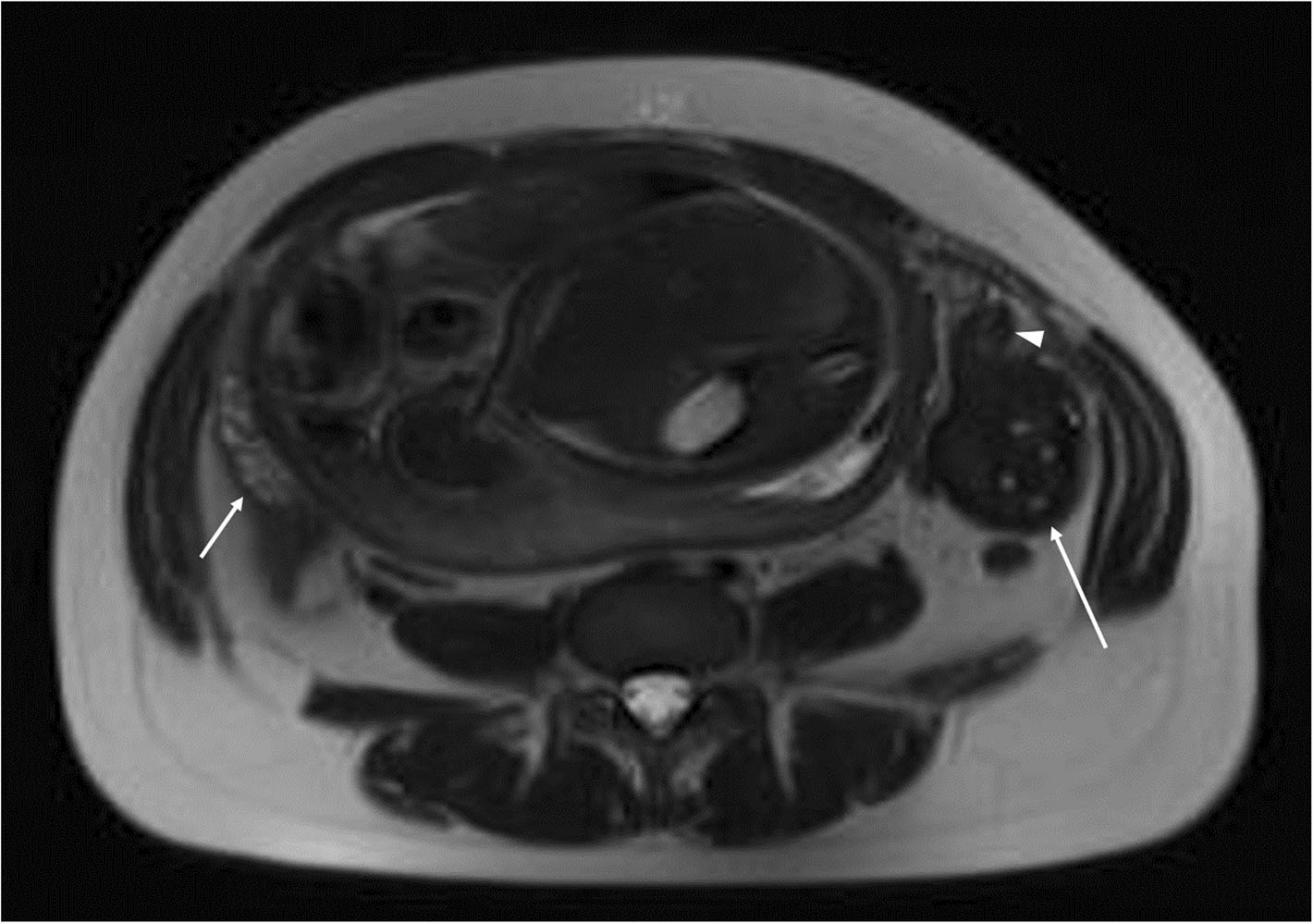
Axial T2-weighted magnetic resonance imaging (MRI) shows an enlarged left ovary (long arrow) with multiple follicles and extensively low signal intensity compared with the normal right ovary (short arrow). The adjacent left fallopian tube was found to be thickened (arrowhead) with the diameter of 11 mm

**Fig. 3:**
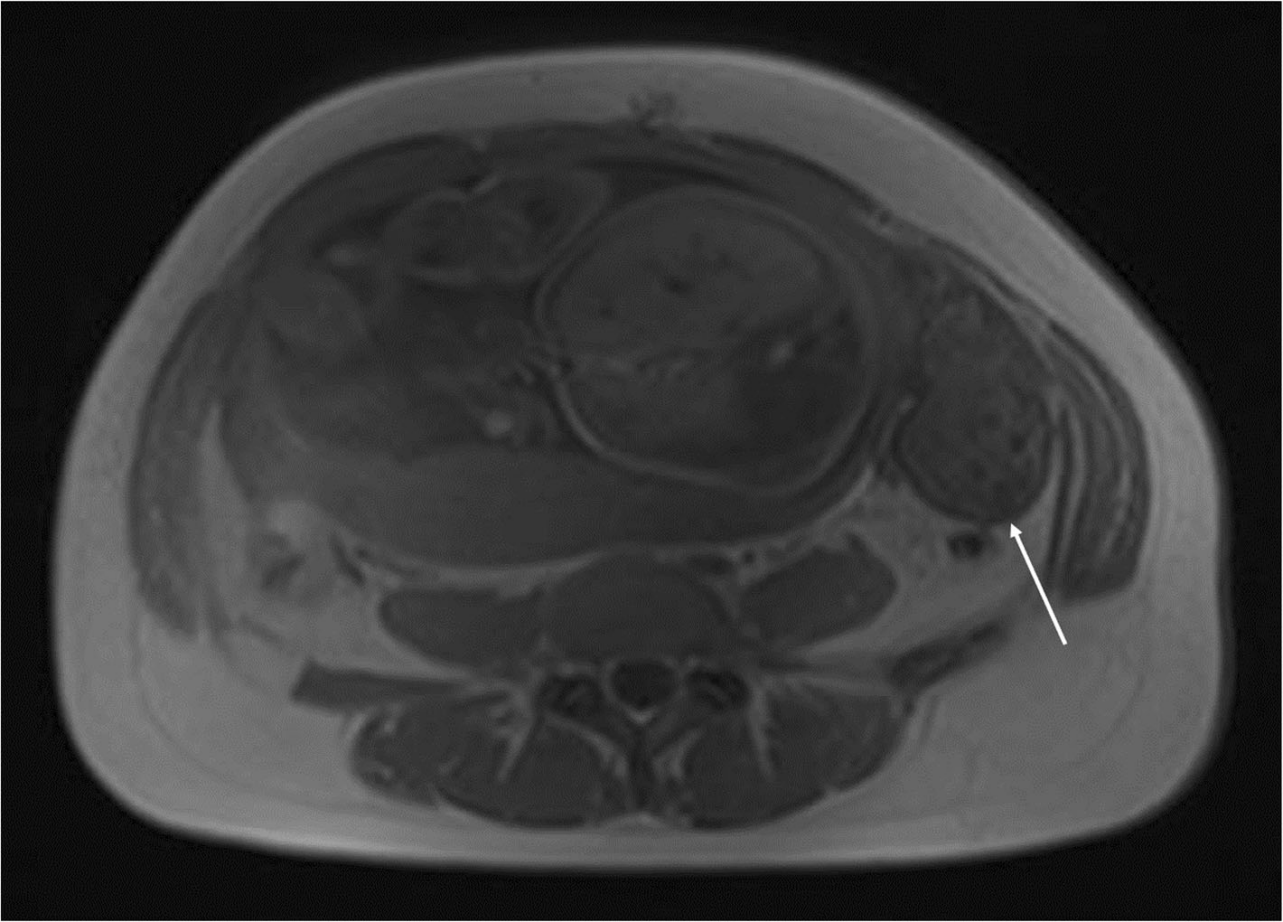
Axial fat-saturated T1 weighted magnetic resonance imaging (MRI) shows the enlarged left ovary had a signal intensity equal to that of muscle but was not homogeneous (long arrow)

To clarify the origin and the nature of the mass and decide the need for immediate surgical intervention, the patient subsequently underwent a prompt MRI scan. MRI showed the left ovary was increased in size to 82 × 42 × 85 mm with peripheral follicles. On fat-suppressed T2-weighted images, the signal intensity of the lesion was significantly decreased (Fig. 2) compared with the right ovary. On fat-saturated T1-weighted images, the enlarged left ovary had a signal intensity equal to that of muscle but was not homogeneous (Fig. 3). The adjacent fallopian tube was thickened, the diameter of the thickened tube was 11 mm (Fig. 2). Its signal intensity was similar to that of the left ovary. A small amount of fluid was detected between the mass and the uterus, as well as in the pouch of Douglas. The normal right ovary was also visualized. Combining the clinical symptoms with the US examination, three radiologists with about 20 years of experience in abdominal MR imaging diagnosed ovary infarction secondary to adnexal torsion.

**Fig. 4:**
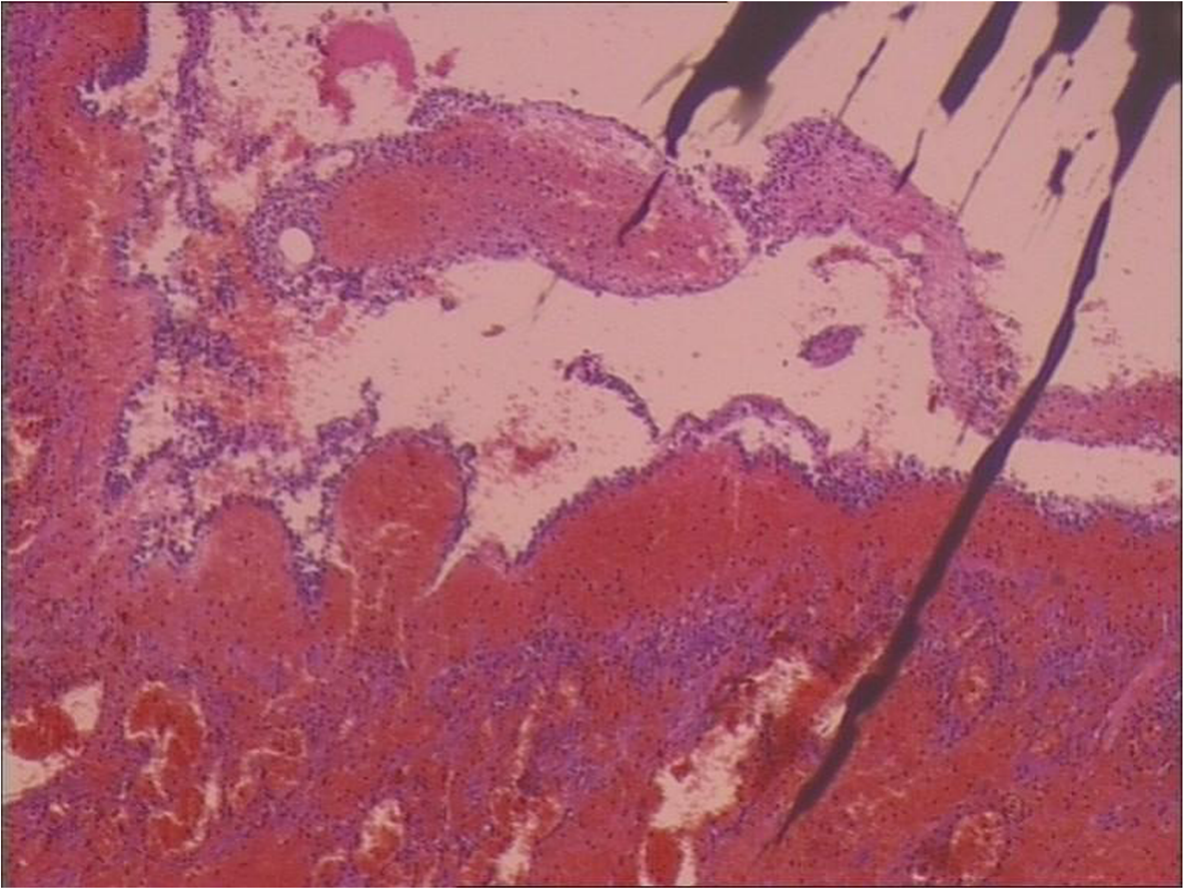
Hemorrhage and necrosis of ovary H&E × 20. H&E: hematoxylin and eosin

With the provisional diagnosis of adnexal torsion, the patient was taken to surgery. During surgery, the left ovary was found to be twisted twice over (720°) around its pedicle and the left tube. The left ovary was 80 × 60 × 50 mm with a purple hue. The right adnexa and uterus were normal. As there was extensive hemorrhage and necrosis, a left salpingo-oophorectomy was performed. The histopathology confirmed an extensively hemorrhagic fallopian tube and ovary with partial necrosis (Fig. 4) and described multiple follicular cysts in left ovary. The pregnancy continued without problems, and the patient was discharged on postoperative day 3.

## Discussion and conclusions

Adnexal torsion is frequently associated with ovarian hyperstimulation therapy or ovarian masses. The torsion of normal adnexa is rare during pregnancy, especially in the third trimester. Early diagnosis and treatment are essential to save the adnexa and decrease maternal and fetal morbidity [8]. Adnexal torsion is difficult to diagnose during pregnancy, because its symptoms and signs are nonspecific and can be confusing when compared with other acute abdominal conditions. In the present case, we found that aside from hyperintensity on fat-saturated T1-weighted images, the low signal intensity on T2-weighted images can also reflect adnexal hemorrhage when the torsion of normal adnexa occurred during pregnancy. This case demonstrates one instance where MRI is valuable in diagnosis of adnexal torsion in advanced gestation with equivocal sonographic findings.

MRI is a useful diagnostic modality when visualizing ovaries by routine US is difficult in the case of adnexal torsion suspected during the second and third trimesters of pregnancy [9]. The specific MRI imaging finding for adnexal torsion is tube thickening, which is related to congestion and edema with or without hemorrhagic infarction of the tube. The tube is considered thickened when the diameter of the tube exceeds 10 mm [10]. Béranger-Gibert et al. [11] reported that this sign was found in 90.0% of women with adnexal torsion. In our case, we found the thickened tube with the diameter of 11 mm. Other useful findings include a small peritoneal effusion and the deviation of the uterus toward the involved side. However, these findings are not specific for adnexal torsion [10]. The MRI images of the adnexa can present varied characteristics in different phases because adnexal torsion is usually involved in a gradually ischemic process over time [12]. Initially, ovarian torsion causes mild ovarian congestion due to the occluded venous flow. MRI shows ovarian enlargement with diffusing stromal edema seen as hyperintensity on T2-weighted images. The ovary then undergoes hemorrhagic infarction, which is noted as hyperintensity on fat-saturated T1-weighted images, indicating nonviable ovary [13]. In our case, histopathology analysis described an extensively hemorrhagic fallopian tube and ovary with partial necrosis (Fig. 4). We did not detect hyperintensity on fat-saturated T1-weighted images in our case (Fig. 3), but extensively low signal intensity was observed on T2-weighted images (Fig. 2). In several similar reports on the torsion of normal adnexa [5–7], the hemorrhagic and necrotic ovaries still appeared hyperintense on T2-weighted images. Our finding differed from these reported cases. We speculate that the discrepancy may be related to different hemorrhage stages or bleeding amount. With the development of hemorrhagic necrosis, the hyperintensity of the stroma due to edema was invisible on T2-weighted images. When ovarian torsion with hemorrhagic necrosis occurred, immediate surgery was considered necessary.

US is a safe, accurate, fast, noninvasive, and inexpensive primary method for detecting adnexal torsion. The sonographic findings of adnexal torsion included the unilateral ovarian enlargement (> 4 cm), uniform peripheral cystic structures, coexistent mass within the affected ovary, free pelvic fluid, lack of arterial or venous flow, and twisted vascular pedicle [14]. The presence of flow on color Doppler imaging dose not exclude torsion diagnosis. Pena et al. [15] reported that 60% of 21 surgically confirmed cases of ovarian torsion were normal on Doppler ultrasonography, leading to a delayed diagnosis up to 2 days. Factors such as increased bowel gas, small field of view (FOV), obesity and anatomical alterations in late pregnancy can hinder the proper visualization of adnexal regions, making the assessment of adnexal torsion difficult. In our case, the ovaries were displaced by the enlarged uterus in late pregnancy and no clear boundaries were found between the left uterine border and left ovary, so the operators were unable to determine whether the mass originated from the left ovary or the uterus and couldn’t diagnose ovarian torsion definitively.

Compared with US, MRI has different potential advantages, such as large FOV, multiplanar imaging, excellent soft tissue contrast, and ability to differentiate blood from other fluids. A study conducted by Béranger-Gibert et al. [11] reported that the accuracy of MRI was higher than 80% to diagnose the adnexal torsion in the context of acute pelvic pain present for less than 4 h. Because it is difficult to obtain an emergent MRI on an unstable gravid patient with acute hemorrhage or pain, the use of MRI is limited in obstetric emergencies. However, the present MRI can be performed in a relatively short time without any specific patient preparation by using fast MR sequences which can decrease the time to approximately 30 min. Maternal and fetal motion artifacts can be significantly reduced with acquisition time under 20 s during one maternal breath-hold [16]. According to our case, we believe that MRI is a useful problem-solving tool in diagnosis of adnexal torsion in advanced gestation with equivocal sonographic findings. Although MRI is more expensive than US, we still recommend that it should be used as a second examination in pregnant women with unclear diagnosis. To some extent, MRI can indicate the developmental stage of adnexal torsion, thereby providing guidance for clinical treatment.

In conclusion, the diagnosis of adnexal torsion in advanced gestation is difficult to make due to nonspecific symptoms and signs and the limitations of US. MRI is helpful where US is indeterminate in diagnosis of the torsion of normal adnexa in advanced pregnancy. MRI is sensitive in detecting adnexal hemorrhage when the torsion of the normal adnexa occurred during pregnancy, and on imaging is displayed as hyperintensity on fat-saturated T1-weighted images or low signal intensity on T2-weighted images.

## Data Availability

Not applicable.

## Abbreviations

FOV: Small field of view
MRI: Magnetic resonance imaging
US: Ultrasound
